# Behavioural Outcomes Following Neonatal Hypoxia-Ischaemia Suggest Psychopathology-Like Phenotypes and Potential Improvement with Tubastatin-A

**DOI:** 10.1192/bjo.2026.11209

**Published:** 2026-06-30

**Authors:** Jon Ander Alart, Antonia Álvarez, Paola Cantalapiedra, Ana Catalán, Daniel Alonso-Alconada

**Affiliations:** 1 University of the Basque Country, Bilbao, Spain; 2 OSI Bilbao-Basurto, Bilbao, Spain

## Abstract

**Aims:**

Perinatal hypoxia–ischaemia (HI) is a major cause of neonatal mortality and, importantly, of long-term neurodevelopmental morbidity. Children who suffered perinatal asphyxia show a higher risk of developing a wide spectrum of psychiatric disorders, alterations that often coexist with sensorimotor deficits and cognitive problems. Therefore, perinatal HI is increasingly recognized not only as a neurological condition but also as a strong developmental risk factor for later psychopathology.

**Objective:**

To evaluate behavioural outcomes closely linked to psychiatric vulnerability (specifically motor function and different memory domains) and to examine possible sex-dependent differences in neonatal rats subjected to a preclinical model of severe HI and treated with the epigenetic modulator Tubastatin-A (Tub-A).

**Methods:**

Seven-day-old (P7) Sprague Dawley rats were randomly assigned to: i) HI (left common carotid artery ligation + 150 min of hypoxia (8%O_2_/92%N_2_); n=18, 10 males, 8 females); ii) HI + Tub-A (25 mg/kg; one i.p. dose immediately after HI and three more doses spread over the next 72 hours; n=18, 9 males, 9 females); iii) Sham (without HI; n=18, 9 males, 9 females). At P8 and P14, the animals underwent righting reflex, negative geotaxis, and front limb suspension tests to analyse their sensorimotor abilities. At P40 and P90, the animals underwent the cylinder test (to measure sensory-motor deficits), novel object recognition (NOR, to analyse the non-spatial memory) and T-maze test (spatial memory and learning ability). These domains are fundamental for adaptive behaviour and are frequently compromised in psychiatric conditions.

**Results:**

In short-term tests, P8 animals in the Tub-A-treated group showed a better motor response in the front limb suspension test (p=0.004), and especially in males (p=0.0033). At P40, the cylinder test revealed differences in females in the HI group and those treated with Tub-A (p=0.044). In the NOR test, a notable improvement was seen in all treated animals (p=0.0001 vs HI), independent of sex (males: p=0.0022; females: p=0.0012). At P90, the improvement in NOR was maintained overall (p=0.0462) and in males (p=0.0004), but not in females. In the T-maze test, Tub-A-treated males had better results (p=0.0404) at P40, whereas this improvement was enhanced at P90 for both sexes (males: p=0.0083; females: p=0.0423).

**Conclusion:**

Perinatal HI-induced long-term behavioural impairments related to psychopathologies may be improved after Tub-A treatment.

